# Effects of Everolimus-Eluting Stents on the Left Ventricular Systolic and Diastolic Functions

**Published:** 2015-04-03

**Authors:** Mohammad Sadegh Parsaee, Maryam Nabati, Naser Saffar, Morteza Taghavi

**Affiliations:** 1 *Mazandaran University of Medical Sciences, Sari, Iran.*; 2 *Department of Cardiology, Faculty of Medicine, Mazandaran University of Medical Sciences, Sari, Iran.*; 3 *Student Research Committee, Faculty of Medicine, Mazandaran University of Medical Sciences, Sari, Iran.*

**Keywords:** Angioplasty, Stents, Coronary restenosis, Echocardiography, Doppler

## Abstract

**Background:** The drug-eluting stent (DES) decreases the rate of coronary restenosis and re-obstruction. The aim of this study was to assess prospectively the effectiveness of the new generation DES on the left ventricular (LV) systolic and diastolic functions in patients with isolated severe proximal left anterior descending (LAD) coronary artery stenosis.

**Methods: **A prospective study was conducted on 50 patients with isolated severe proximal LAD stenosis. Successful percutaneous coronary intervention (PCI) with Everolimus-eluting stents was performed for the whole study population. All the patients underwent transthoracic echocardiography within 24 hours before and one month after PCI, and LV systolic and diastolic parameters were compared before and after PCI using the paired samples t-test.

**Results: **The mean age of the study population was 57.68 ± 8.82 years. Within the study population, 26 (52%) patients were male and 24 (48%) were female. There was a significant 10.6% and 5.2% increase in the early diastolic mitral annular motion (e') and the LV ejection fraction following PCI, respectively (p value = 0.005 and p value = 0.044, respectively). Before PCI, wall motion abnormality was seen in 2.21 ± 2.91 segments, which significantly decreased to 1.49 ± 2.58 segments (p value = 0.04) after the procedure. Also, the wall motion score index was 1.18 ± 0.26 before PCI, which significantly decreased to 1.13 ± 0.23 after PCI (p value < 0.001).

Also, there was a trend toward a higher ratio of transmitral peak early diastolic velocity to peak late diastolic velocity after PCI (p value = 0.068).

**Conclusion:** Our study showed that the use of the Everolimus-eluting stents improved the LV systolic and diastolic functions in patients with isolated severe LAD stenosis.

## Introduction

Lesions in the proximal segment of the left anterior descending (LAD) coronary artery present a high-risk lesion, as this important anatomical location of atherosclerotic plaques is associated with increased mortality.^1^ Indeed, the LAD supplies 40-50% of the total left ventricular (LV) myocardium. Therefore, obstruction at this site causes ischemia in a large portion of the myocardium.^2^

New angioplasty techniques have led to higher success rates of percutaneous coronary intervention (PCI), and drug-eluting stents have decreased the rate of restenosis and re-obstruction.^3^ Echo Doppler studies have reported that patients with asymptomatic LV diastolic dysfunction have a higher incidence rate of all-cause mortality. Mild diastolic dysfunction and moderate-to-severe dysfunction were associated with 8.3-fold and 10.2-fold increased risks of mortality, respectively. The overall mortality rates of symptomatic patients with diastolic or systolic heart failure are very similar.^4^

Transmitral Doppler flow velocities have previously been used for evaluating the variations in the LV diastolic function after PCI.^5^ The left atrial diameter, early filling deceleration time (DT), mitral annular early diastolic velocity (e'), ratio of transmitral peak early diastolic velocity to mitral annular early diastolic velocity (E/e'), and peak atrial reversal flow velocity of the pulmonary veins are relatively independent of preload and are a more reliable index of the LV relaxation. Additionally, the LV ejection fraction (LVEF) and peak systolic velocity (s') of the mitral annulus have been used as indices of the global LV systolic function.

Therefore, our study was designed to determine the changes in the LV systolic and diastolic functions after successful PCI and Everolimus-eluting stenting of isolated stenosis of the proximal LAD.

## Methods

From 2012 to 2013, a prospective clinical study was conducted in Fatemeh Zahra teaching hospital. In total, 50 consecutive patients were considered for enrollment who presented with typical chest pain and documented myocardial ischemia or previous non-ST-segment elevation myocardial infarction with a considerable amount of anterior or anteroseptal ischemia on *single-photon emission computerized tomography* and who underwent successful angioplasty after displaying isolated severe proximal LAD stenosis (> 75% luminal diameter) in a recent angiography. The diagnosis of non-ST-segment elevation myocardial infarction was based on the elevation in cardiac biomarkers (creatine kinase MB or troponin) and evidence of ischemia based on symptoms, electrocardiography, or imaging. 

The study was performed according to the guidelines of the Helsinki Declaration and was approved by the ethics committee of the hospital. Written informed consent was obtained from all the patients.

Patients with lesions in the first diagonal branch, total occlusion of the LAD, or multivessel coronary artery disease were excluded from our study. None of the study patients had congenital, significant valvular heart disease (≥ moderate severity) or cardiomyopathy. Additionally, patients with systemic diseases such as cancer, collagen vascular diseases, and amyloidosis were excluded.

Transthoracic echocardiography was performed at baseline within 24 hours before PCI and was repeated one month after PCI for all the patients using a Vivid S5 (GE Healthcare, Wauwatosa, WI, USA), 1-3 MHz transducer. All the measurements represent the average of three consecutive beats within the normal heart rate range, 60 - 100 beats per minute. The images were stored on a hard disc for better offline measurements, and the results were confirmed by an echocardiographer, who was blind to the patient’s information. Patients with a poor echo window were excluded from the study.

To assess the reproducibility of the echocardiographic measurements, these indices were measured (according to systematic sampling method) in 10 randomly selected patients and were repeated one day later to calculate the intra-observer correlation coefficients, which was found to be 0.9.

Estimates of the LV systolic and diastolic dimensions were derived from the LV minor-axis dimensions with the transducer in the parasternal position so that the cursor was perpendicular to the interventricular septum and posterior wall at mid-papillary muscle level. The ejection fraction (EF) and wall motion abnormalities (WMA) were determined. The EF was defined as the end-diastolic volume (EDV) minus the end-systolic volume (ESV) divided by the EDV from the biplane apical two- and four-chamber views using a modified Simpson technique. Also, the wall motion score index (WMSI) was determined. The total score was derived by assigning a grade of 1 through 5 (normal = 1, hypokinetic = 2, akinetic = 3, dyskinetic = 4, and aneurysmal = 5). The scores for each segment were added and then divided by the number of segments graded for a total wall motion score.^6^ Pulse Doppler recordings of the diastolic transmitral flow velocity were obtained with the sample volume located at the tips of the mitral leaflets from the apical four-chamber view.

The peak early diastolic velocity (E wave), peak late diastolic velocity (A wave), early filling deceleration time (DT), and the peak early diastolic velocity/peak late diastolic velocity (E/A) were measured. Tissue Doppler imaging of the mitral annulus was obtained from the apical four-chamber view. A 5-mm sample volume was placed at the septal and lateral mitral annuli.

The following measurements were determined: the peak systolic velocity (s') and early diastolic velocity (e'). An analysis was performed for the average of each velocity at the two annular sites. Then, the E/e' ratio was calculated. The pulmonary venous flow velocity profile was obtained from an apical four-chamber view. The pulse Doppler sample volume was placed 1-2 cm into the pulmonary vein, and the peak systolic flow velocity (S wave), peak diastolic velocity (D wave), and peak atrial reversal flow velocity (AR) were determined. The left atrial diameter was measured in the parasternal long- axis view from a two-dimensional image at end systole.

Blood samples were obtained during fasting, and the levels of plasma glucose, total cholesterol, high-density lipoprotein cholesterol, Low-density lipoprotein cholesterol, and triglycerides were measured**.** The systolic and diastolic blood pressures were measured after 5 minutes of rest. The height and weight were measured, and the body mass index was calculated as the body weight divided by the height squared. Hypertension was defined as a systolic blood pressure ≥ 140 mmHg, a diastolic blood pressure ≥ 90 mmHg,^7^ or requirement for antihypertensive medication. Diabetes mellitus was defined according to the criteria of the American Diabetes Association or requirement for insulin or oral hypoglycemic drugs.^8^ A family history of coronary artery disease was defined as having a first-degree relative (a male < 55 years old or a female < 65 years old) with a history of myocardial infarction, coronary revascularization, or sudden death.^9^ The history of smoking was determined via a face-to-face questionnaire. Coronary angiography was performed for all the patients using a cardiac angiography system (Siemens AG, Medical Solutions, Erlangen, Germany), and they all underwent PCI. PCI was performed via standard techniques, and XIENCE stents (Everolimus-Eluting Coronary Stent System, 3200 Lakeside Drive, Santa Clara, CA 95054 USA) were used. Procedural success was defined as the successful deployment of the stent and residual stenosis < 30%.^10^ Procedural anticoagulation was achieved with unfractionated Heparin; glycoprotein IIb/IIIa inhibitors were used whenever needed (intravenous Eptifibatide). The patients received 300 mg of Aspirin before the intervention. A 300 mg oral dose of Clopidogrel was recommended before the procedure. Thereafter, 80 mg of Aspirin and 75 mg of Clopidogrel were prescribed daily. Other standard drugs (angiotensin-converting enzyme inhibitors, beta blockers, statins, and oral hypoglycemic agents) remained unchanged during the study in order to minimize the effects of alterations on the echocardiographic variables. During the follow- up period, all the patients were asymptomatic. 

The continuous variables are expressed as mean ± SD. The paired samples t-test was used to compare the means of the variables before and after PCI. A p value < 0.05 was considered statistically significant.

For the statistical analyses, the statistical software SPSS version 18.0 for Windows (SPSS Inc., Chicago, IL) was used.

## Results

A total of 50 patients were enrolled in the study. The mean age of the study population was 57.68 ± 8.82 years. Within the study population, 26 (52%) patients were male; 23 (46%) were diabetic; 8 (16%) were smokers; 15 (30%) had a positive family history of coronary artery disease; and 34 (68%) were hypertensive. Ten (20%) patients were admitted with non-ST-segment myocardial infarction. The other demographic and measures of common cardiovascular risk factors of the study population are presented in Table 1.

Before performing PCI, the percentage diameter stenosis was 89.29 ± 11.17%. Again, after performing PCI, it decreased to 5.07 ± 6.17% (p value < 0.001). Additionally, the stent diameter was 2.81 ± 0.27 mm; the stent length was 24.41 ± 7.86 mm; and the stent number was 1.05 ± 0.23. The changes in the echocardiographic indices of the LV systolic and diastolic functions following coronary stenting were assessed via the paired samples t-test.

Table 2 compares the echocardiographic indices of the LV systolic and diastolic functions before and after PCI.

PCI created a statistically significant increase in the LVEF (5.28%) and e' velocity (10.49%). Before PCI, the WMA was observed in 2.21 ± 2.91 segments, which significantly decreased to 1.49 ± 2.58 segments (p value 0.039) after the procedure. Also, the WMSI was 1.18 ± 0.26 before PCI, which significantly decreased to 1.13 ± 0.23 after PCI (p value < 0.001). 

Also, there was a trend toward a higher E/A ratio after PCI (p value = 0.068). Both, diastolic and systolic left ventricular internal dimension (LVIDs) decreased from 4.73 ± 0.41 cm and 2.97 ± 0.53 cm to 4.69 ± 0.51 cm and 2.95 ± 0.64 cm, correspondingly. These differences were not statistically significant. Furthermore, the end-diastolic and systolic LV volumes decreased from 103.75 ± 10.12 cm^3^ and 43.72 ± 5.32 cm^3^ to 93.25 ± 9.85 cm^3^ and 30.95 ± 5.12 cm^3^, respectively. These differences were not statistically significant (p value = 0.077 and 0.128, respectively). Also, the s' velocity increased from 6.91 ± 1.36 cm/s to 7.09 ± 1.32 cm/s, which was not statistically significant. The ratios of S/D and atrial reversal velocity were unchanged.

**Table1:** Patients' characterises (n=50)

	Mean	Std. Deviation
Age (y)	57.68	8.83
Height (cm)	166.61	8.28
Weight (kg)	74.38	12.72
BMI (kg/m^2^)	26.77	3.94
FBS (mg/dl)	144.23	79.25
TG (mg/dl)	212.69	128.79
CHOL (mg/dl)	184.84	50.21
HDL (mg/dl)	43.89	18.06
LDL (mg/dl)	102.01	21.05

BMI, Body mass index; FBS, Fasting blood sugar; TG, Triglyceride; CHOL, Cholesterol; HDL, High-density lipoprotein; LDL, Low-density lipoprotein

**Table 2:** Echocardiographic indices of the left ventricular systolic and diastolic functions before and after PCI

	Before PCI	After PCI	Difference (after-before)	P value
LVIDd (cm)	4.73±0.41	4.69±0.51	-0.03 (-0.63%)	0.689
LVIDs (cm)	2.97±0.53	2.95±0.64	-0.04 (-1.34%)	0.718
LA (cm)	3.59±0.41	3.68±0.42	0.09 (2.51%)	0.116
EF (%)	50.35±9.19	53.01±9.42	2.66 (5.28%)	0.044
DT (ms)	274.24±65.64	271.38±90.38	-2.86 (-1.04%)	0.831
E/A	0.82±0.27	0.90±0.31	0.08 (9.75%)	0.068
S/D	1.49±0.34	1.49±0.41	-0.001 (-0.01%)	0.984
Areversal (cm/s)	35.98±7.68	34.80±6.41	-1.18 (-3.28%)	0.432
e'average (cm/s)	8.01±2.26	8.85±2.39	0.84 (10.49%)	0.005
E/e'	8.11±2.71	7.87±2.27	-0.23 (-2.83%)	0.487
s' average (cm/s)	6.91±1.36	7.09±1.32	0.19 (2.75%)	0.515

PCI, Percutaneous coronary intervention; LVIDd, Diastolic left ventricular internal dimension; LVIDs, Systolic left ventricular internal dimension; LA, Left atrium; EF, Ejection fraction; DT, Early filling deceleration time; E/A, Transmitral peak early diastolic velocity/peak late diastolic velocity; S/D, Pulmonary vein peak systolic/peak diastolic velocity; E/e', Transmitral peak early diastolic velocity/mitral annular early diastolic velocity; s', Mitral annular peak systolic velocity

## Discussion

Regarding revascularization strategies for isolated proximal LAD stenosis, the results suggest that coronary artery bypass graft surgery and PCI are comparable, although with a much higher rate of repeat revascularization within the first years with bare-metal stenting. Data for drug-eluting stents are lacking.^11^

Polymer-based Paclitaxel-eluting stents and Sirolimus-eluting stents have been shown to significantly reduce angiographic restenosis in comparison with bare-metal stents. However, the rates of primary stent thrombosis are increased. With the goal of further augmenting the security and effect of the drug-eluting stent, an Everolimus-eluting stent has been designed.^12^

In a 14-day rabbit iliac model, endothelialization over the stent struts was more rapid with the Everolimus-eluting stent than with Sirolimus-, Zotarolimus-, or Paclitaxel-eluting stents.^13^ In 2000, S. Bayata^5^ evaluated the early effect of PCI on the LV diastolic dysfunction in 30 patients with isolated severe LAD stenosis. All the measurements were performed within 4 hours before PCI and repeated within 24 hours after angioplasty. After angioplasty, none of the parameters (DT, isovolumic relaxation time (IVRT), E/A, and E wave transit time), except for the A-wave transit time, was changed significantly. However, the tissue Doppler imaging (TDI) study and the calculation of the EF as well as the LV and left atrium were not included in this study. Additionally, stenting was not performed for all the patients. 

Previous studies have shown a close inverse correlation between the ratio of transmitral peak early velocity to peak late diastolic velocity and the pressure-derived variables of the LV relaxation. Also, there is compensatory augmentation of the atrial filling in patients with impaired LV relaxation.^14^ Impaired LV relaxation is the earliest manifestation of myocardial ischemia. The primary abnormality of myocardial relaxation is characterized by a decrease in the E wave velocity, increase in the A-wave velocity, decrease in the E/A ratio, and increase in the DT and AR velocity. Later, it is followed by an increase in the left atrial diameter. Additionally, a decrease in the early diastolic mitral annular motion (e') is related to a decreased LV elastic recoil, which is noted as an early sign of ischemia and impaired relaxation.

In our study, the percentage increase in the ratio of the peak early diastolic velocity to the peak late diastolic velocity after PCI was 9.75% (a trend toward significance). These data suggest, but do not prove, that PCI on the LAD may be accompanied by improvement in the LV relaxation. Also, there was a significant 10.6% increase in the early diastolic mitral annular motion due to improvement in the LV elastic recoil. In contrast, the LV systolic function is an important predictor of prognosis, and parameters that represent the systolic function such as the LVEF, WMA, and systolic and diastolic LV internal dimensions can be used for predicting outcomes.^15^

We detect a significant 5.26% increase in the LVEF following PCI as an important marker of reperfusion and improved outcome. Also, there was significant improvement in the WMA and WMSI after the procedure. Using the E/e' ratio, a ratio < 8 is usually associated with normal LV filling pressures, whereas a ratio > 15 is associated with increased filling pressures. When the value is between 8 and 15, other echocardiographic indices should be used. In our study, the majority of the patients had impaired relaxation with a normal filling pressure (E/e' = 8.1 ± 2.7) before PCI. It can be an explanation for the absence of a significant change in the E/e' ratio following PCI.^16^

Our study showed that Everolimus-eluting stents may be a good selection in patients with isolated severe proximal stenosis of the LAD. These stents favorably improved the markers of the LV systolic function such as the LVEF. Also, there was significant improvement in the early diastolic mitral annular motion as a marker for improved LV elastic recoil and diastolic function.

A limitation of our study was the small sample size. Non-significant differences between the S/D (pulmonary vein peak systolic/ peak diastolic velocity), E/e' (trans mitral peak early diastolic velocity / mitral annular early diastolic velocity) , AR (pulmonary vein atrial reversal velocity), left atrial diameter, s' velocity (mitral annular peak systolic velocity), LVIDs (systolic left ventricular internal dimension, LVIDd (diastolic left ventricular internal dimension), LVEDV (left ventricular end diastolic volume), and LVESV (left ventricular end systolic volume) before and after PCI may be due to the small sample size. Another limitation was the use of the left atrial diameter instead of the volume. Additionally, our follow- up period was only one month after PCI. Therefore, a longer follow-up period in future studies may produce more consistent results.

## Conclusion

Our study showed that the use of the Everolimus-eluting stents improved the LV systolic and diastolic functions in patients with isolated severe LAD stenosis. 

## Notes:

***This paper should be cited as:*** Parsaee MS, Nabati M, Saffar N, Taghavi M. Effects of Everolimus-Eluting Stents on the Left Ventricular Systolic and Diastolic Functions. J Teh Univ Heart Ctr 2015;10(2):93-97.
